# Special Issue “Pathogenesis and Treatments of Head and Neck Cancer”

**DOI:** 10.3390/ijms262412107

**Published:** 2025-12-16

**Authors:** Marko Tarle

**Affiliations:** 1 Department of Maxillofacial Surgery, Dubrava University Hospital, 10000 Zagreb, Croatia; tarlemarko1@gmail.com; 2 School of Dental Medicine, University of Zagreb, Gundulićeva 5, 10000 Zagreb, Croatia

Head and neck cancers (HNCs) are among the most common malignancies worldwide and represent a substantial global health burden. Recent GLOBOCAN estimates report nearly 950,000 new cases and approximately 480,000 deaths each year [1,2]. Although age-standardized incidence and mortality rates have changed only modestly in recent decades, the absolute number of cases continues to rise due to population growth and aging [3,4]. Beyond these global trends, the epidemiology of HNC is undergoing important qualitative shifts that challenge traditional risk-based and anatomical classifications. HPV-positive oropharyngeal squamous cell carcinomas (OPSCC) have now surpassed cervical cancer as the most common HPV-associated malignancy in the United States, a shift driven largely by a sharp rise in HPV-positive OPSCC among men [5,6]. Most of these tumors originate in the crypt epithelium of the palatine tonsils and the base of the tongue, where the reticulated mucosa favors persistent HPV infection [7]. Men are disproportionately affected, exhibiting higher oral HPV prevalence, longer infection persistence, and substantially lower vaccination coverage than women [8]. The causes of this disparity, such as potential differences in mucosal immunity, patterns of oral HPV exposure, or unidentified biological factors, remain insufficiently explained and require focused investigation [9]. These observations emphasize that viral oncogenesis in the oropharynx cannot be fully understood without integrating host immune regulation and microenvironmental context.

In Europe, particularly in the United Kingdom, there has been a steep rise in oral cavity cancer incidence, with an increase of more than 130% over the past two decades [10]. Earlier analyses showed a particularly sharp rise among patients younger than 45 years, while more recent data highlight a continued increase in tongue cancers among young nonsmoking women, suggesting the possible role of yet unidentified etiological factors beyond the classical ones of tobacco, alcohol, and HPV [11,12]. Together, these epidemiological patterns point toward a biologically heterogeneous disease spectrum that cannot be adequately captured by exposure history alone. In this context, the upcoming ninth edition of the TNM classification will further refine staging, especially in HPV-positive oropharyngeal carcinomas. The revision will more precisely define depth of invasion in oral cavity cancers and extranodal extension in neck metastases, while moving toward the integration of biological parameters that increasingly determine prognosis and treatment outcomes [13]. This evolution of staging frameworks mirrors a broader conceptual shift: HNC is increasingly viewed not as a uniform entity but as a constellation of biologically distinct diseases. The clinical and biological heterogeneity of HNC, traditionally described as two “faces” of the disease, one associated with cumulative tobacco and alcohol exposure and the other occurring in younger individuals without classical risk factors, underscores the need for an integrative models that combine epidemiology with molecular and immunological determinants. These changing disease patterns have accelerated interest in the role of immune dysregulation, chronic inflammation, and microbiome imbalance as central drivers of carcinogenesis and disease progression.

There is now ample evidence that immune dysregulation, microbiota imbalance, and chronic inflammation together create a microenvironment conducive to carcinogenesis. Oral dysbiosis and periodontitis drive oxidative stress, epigenetic remodeling, and tumor immune tolerance [14,15,16]. The role of regulatory T cells (T-regs) is particularly intriguing, as they can suppress antitumor immunity but also protect against excessive inflammatory toxicity. This duality highlights a central dilemma in HNC immunobiology: how immune suppression and immune protection coexist within the same tumor ecosystem. The Nobel Prize in Medicine 2025, awarded to Sakaguchi, Ramsdell, and Brunkow for the discovery of FOXP3-mediated peripheral immune tolerance, highlights how a refined understanding of immune equilibrium can lead to therapeutic strategies that modulate immunity, either by selectively dampening T-reg activity in tumors or by developing immunopreventive interventions for high-risk populations [17,18].

The treatment of HNC is rapidly evolving toward a precision-based, minimally invasive, and biologically guided approach. While surgery, radiotherapy, and chemotherapy remain the cornerstones of curative management, major advances have reshaped their use [19]. Immunotherapy, particularly PD-1/PD-L1 inhibitors such as pembrolizumab and nivolumab, has become the standard of care for recurrent and metastatic disease, providing durable responses in a subset of patients [20]. Immunotherapy is increasingly used in earlier disease stages. Neoadjuvant and perioperative administration have shown encouraging results, including high rates of major pathological response and improved disease control, raising the possibility of reducing the intensity of adjuvant treatment and better tailoring therapy to tumor biology [21,22]. These approaches raise important questions regarding treatment de-escalation and the biological selection of patients most likely to benefit. An important unresolved question remains how emerging biological stratification can be translated into clinically actionable decision-making without increasing treatment complexity or patient burden. Surgical practice has been transformed by transoral robotic surgery (TORS) and transoral laser microsurgery (TLM), which allow precise tumor resection with superior functional outcomes and reduced morbidity, particularly in HPV-positive oropharyngeal cancers, where treatment de-escalation strategies are gaining momentum [23]. At the same time, artificial intelligence (AI) is entering clinical practice through enhanced imaging interpretation, automated diagnostics, prognostic modeling, and personalized treatment planning, supporting earlier detection and adaptive radiotherapy [19,24].

The thirteen studies in this Special Issue are situated at the intersection of immunology, genetics, microenvironmental biology, and therapy. Rather than addressing isolated molecular targets, these contributions collectively conceptualize HNC as a dynamic ecosystem shaped by interconnected genetic, metabolic, microbial, and immunological processes. Several studies focus on signaling pathways and invasive behavior, identifying potentially actionable vulnerabilities such as Hedgehog pathway activation in sinonasal adenocarcinoma and the role of aspartate β-hydroxylase in regulating tumor invasiveness (contributions 1 and 2). These findings reinforce the notion that key therapeutic leverage points may reside in pathway-level and metabolic regulation rather than single oncogenic drivers. From a therapeutic perspective, experimental evidence demonstrates that combined inhibition of DNA-PKcs and PARP produces robust radiosensitization in HNSCC cell lines while sparing normal fibroblasts (contribution 3), supporting the rationale for biologically guided combinations with precision radiotherapy. Diagnostic and prognostic advances are addressed through the identification of keratin expression patterns associated with tumor margins and HPV status (contribution 4), as well as transcriptomic signatures in laryngeal dysplasia that distinguish progressive from nonprogressive lesions based on immunogenetic features (contribution 5). These studies collectively highlight the emerging role of early immune-related biomarkers in risk stratification and surveillance.

The tumor microenvironment emerges as a central determinant of outcome. Elevated expression of immune checkpoint molecules such as PD-1, LAG-3, and TIM-3 correlates with improved survival, particularly in HPV-positive tumors (contribution 6), suggesting that checkpoint expression may also reflect a structured and potentially exploitable antitumor immune response. This observation challenges simplistic interpretations of immune exhaustion and supports the development of combinatorial immunotherapeutic strategies. The importance of immunometabolism is further underscored by studies demonstrating the prognostic impact of IL4I1 and IDO1 expression (contribution 7) and by evidence that necrotic tumor cells release endogenous danger signals capable of activating Toll-like receptor 3 and promoting protumorigenic signaling (contribution 8). Intratumoral microbiome analysis identifies specific bacterial genera associated with improved survival and activation of oncogenic signaling pathways (contribution 9), suggesting that microbial balance may modulate tumor behavior and therapeutic responsiveness. Metabolic reprogramming is further implicated in treatment resistance, with high expression of SLC2A3 and SDHA predicting local recurrence after radiotherapy or chemoradiotherapy (contribution 10). These convergent findings position immunometabolism and microbiome interactions as promising, yet still underexplored, therapeutic targets.

Together, these contributions present a coherent view of HNC as a biologically complex and evolving disease in which genetic, immunological, metabolic, and microbial factors intersect. This Special Issue illustrates how contemporary HNC research is moving beyond descriptive classification toward integrative, mechanism-driven frameworks. As the forthcoming ninth TNM edition offers a more precise anatomical staging system, molecular and immunological insights increasingly complement clinical decision-making. Taken together, this Special Issue highlights that future advances in HNC management will depend on integrative strategies that align epidemiology, immune biology, microbiome research, and biologically guided therapy within a personalized treatment paradigm.
